# Serum Perilipin-2 as a Novel Biomarker for Obstructive Sleep Apnea: Association with Hypoxic Burden and Disease Severity

**DOI:** 10.3390/jcm15051776

**Published:** 2026-02-26

**Authors:** Gulseren Sagcan, Hafize Uzun

**Affiliations:** 1 Department of Chest Diseases, Faculty of Medicine, Acibadem Mehmet Ali Aydinlar University, Altunizade Hospital, Istanbul 34662, Turkey; 2 Department of Medical Biochemistry, Faculty of Medicine, İstanbul Atlas University, Istanbul 34403, Turkey; huzun59@hotmail.com

**Keywords:** obstructive sleep apnea, perilipin-2, inflammation, apnea–hypopnea index, body mass index

## Abstract

**Background:** Obstructive sleep apnea (OSA) syndrome is a common sleep-related breathing disorder characterized by recurrent upper airway collapse during sleep and is closely associated with metabolic dysregulation, including insulin resistance, adipose tissue dysfunction, and impaired lipid metabolism. Perilipin-2 (PLIN-2), a lipid droplet-associated protein involved in triglyceride storage and regulation of lipolysis, may reflect alterations in lipid homeostasis associated with OSA. **Objective:** This study aimed to evaluate the association between serum PLIN-2 levels and OSA and to assess the relationship between PLIN-2 concentrations and disease severity. **Methods:** A total of 231 participants were included in this study, comprising 70 healthy controls and 161 patients with OSA. Patients were classified according to apnea–hypopnea index (AHI) as having mild (*n* = 60), moderate (*n* = 52), or severe OSA (*n* = 49). All participants underwent overnight polysomnography (PSG). **Results:** Serum PLIN-2 levels were significantly higher in patients with OSA and increased progressively with disease severity. PLIN-2 levels were positively correlated with polysomnographic indices of OSA severity, including AHI and oxygen desaturation index. ROC analysis demonstrated good discriminative performance of PLIN-2 for OSA presence and for distinguishing mild from severe OSA. **Conclusions:** This study is the first to demonstrate an association between serum PLIN-2 levels and OSA. Our findings suggest that PLIN-2 may serve as a novel biomarker reflecting metabolic and lipid-related disturbances in OSA and may provide new insights into the pathophysiological link between intermittent hypoxia and altered lipid metabolism.

## 1. Introduction

Obstructive sleep apnea (OSA) syndrome is defined by recurrent episodes of upper airway collapse, leading to transient hypoxemia, brief nocturnal arousals, and a subsequent disruption of sleep architecture. OSA ranks among the most prevalent sleep disorders [1]. The prevalence of OSA ranges from 6% to 17% and is associated with age, male gender, and obesity, contributing to cardiovascular morbidity and deaths [2,3]. The two primary forms of sleep apnea are obstructive and central. Central sleep apnea (CSA) arises from impaired respiratory control, whereas OSA results from upper airway collapse despite preserved respiratory drive. The pathophysiology of OSA involves inadequate activity of upper airway dilator muscles, leading to airway narrowing or closure during sleep under the influence of negative inspiratory pressure [4,5,6].

OSA is a systemic disorder that is strongly associated with metabolic disturbances, including adipose tissue dysfunction, insulin resistance, and dysregulated lipid metabolism. These alterations are largely driven by intermittent hypoxia and chronic low-grade inflammation, which play central roles in OSA pathophysiology Growing evidence suggests that OSA-related metabolic dysregulation extends beyond traditional risk factors, highlighting the need to identify novel biomarkers that reflect underlying adipose tissue and lipid metabolic alterations [7].

Perilipin (PLIN) family proteins are lipid droplet-associated proteins that play a central role in the regulation of cellular lipid metabolism. In mammalian cells, five PLIN isoforms (PLIN 1–5) have been identified. PLIN-2, also known as adipophilin or adipose differentiation-related protein (ADRP), is ubiquitously expressed and localized on the surface of lipid droplets, where it facilitates lipid droplet formation and stabilizes triglyceride storage. By limiting the access of lipolytic enzymes, PLIN-2 protects triglycerides from hydrolysis and reduces the release of free fatty acids and glycerol, thereby contributing to lipid homeostasis [8,9,10,11,12,13,14].

Intermittent hypoxia, a hallmark of OSA, induces oxidative stress, systemic inflammation, and adipose tissue remodeling, all of which are known to disrupt lipid droplet dynamics and intracellular triglyceride handling. PLIN-2, a lipid droplet-associated protein, plays a central role in regulating triglyceride storage and protecting lipid droplets from lipolysis under metabolic stress conditions. Experimental evidence suggests that hypoxia and inflammatory signaling pathways can upregulate PLIN-2 expression as part of cellular adaptive responses to metabolic imbalance. Therefore, PLIN-2 may represent a mechanistic link between hypoxia-induced metabolic dysfunction and systemic inflammation in OSA. However, despite the well-established metabolic consequences of OSA, the relationship between circulating PLIN-2 levels and OSA has not been previously investigated, representing a notable gap in the current literature. Therefore, we hypothesized that serum PLIN-2 levels are altered in patients with OSA and are associated with disease presence and severity. The aim of this study was to investigate, for the first time, the association between serum PLIN-2 levels and OSA and to assess their relationship with polysomnographic indices of disease severity.

## 2. Materials and Methods

### 2.1. Subjects and Study Design

This prospective study was conducted after ethics approval. Participants were consecutively enrolled between 9 January 2026 and 22 January 2026. The study protocol was approved by the Clinical Research Ethics Committee of Acıbadem University (Approval No.: ATADEK-2026/01; Date: 8 January 2026). All procedures were conducted in accordance with the ethical principles of the Declaration of Helsinki, and written informed consent was obtained from all participants prior to their inclusion in the study.

This cross-sectional observational study was carried out at a tertiary sleep disorders center at Acıbadem University, where a total of 231 adult participants were consecutively enrolled. The study population was categorized into two groups: the OSA group (*n* = 161), consisting of patients diagnosed with OSA via polysomnography (PSG), and the control group (*n* = 70), comprising healthy individuals without clinical or objective evidence of sleep-disordered breathing.

#### 2.1.1. Inclusion Criteria

Participants were eligible for inclusion in the study if they met all of the following criteria:

(i) Age ≥ 18 years; (ii) Underwent overnight, attended PSG in a sleep laboratory; (iii) Diagnosed with OSA syndrome based on standard criteria; (iv) Classification of OSA severity according to the AHI as mild, moderate, or severe; (v) Ability to provide written informed consent; (vii) Availability of complete clinical, biochemical, and polysomnographic data.

#### 2.1.2. Exclusion Criteria

(i) Central sleep apnea or predominant central apneic events; (ii) Prior diagnosis or current treatment of sleep-disordered breathing (e.g., continuous positive airway pressure, oral appliance therapy, or upper airway surgery); (iii) Acute or chronic inflammatory diseases, active infection, or autoimmune disorders; (iv) Known malignancy; (v) Chronic liver disease, chronic kidney disease, or advanced heart failure; (vi) Uncontrolled endocrine disorders (including uncontrolled type 2 diabetes mellitus or thyroid disease); (vii) Use of medications known to significantly alter lipid metabolism or inflammatory status if initiated, discontinued, or dose-adjusted within the previous 3 months.

Participants receiving stable long-term therapy (≥3 months without dose modification) with lipid-lowering agents (e.g., statins, ezetimibe, fibrates), antidiabetic medications (e.g., metformin, insulin, GLP-1 receptor agonists, SGLT2 inhibitors), or antihypertensive agents were allowed to participate, provided that their treatment regimen had remained unchanged during the 3 months preceding enrollment.

(viii) Pregnancy or lactation; (ix) Inability to complete polysomnography or provide informed consent.

#### 2.1.3. Healthy Control Subjects Were Required to Have

(i) AHI < 5 events/hour on overnight PSG; (ii) No history of sleep-disordered breathing; (iii) No chronic systemic, metabolic, or inflammatory disease; (iv) Not receiving medications affecting lipid metabolism.

### 2.2. Experimental Procedures

#### 2.2.1. OSA Parameter Assessments

All participants underwent overnight, attended PSG conducted in a sleep laboratory using a standardized montage. Polysomnographic recordings included electroencephalography, electrooculography, submental and tibial electromyography, electrocardiography, airflow measurement via nasal pressure transducer and oronasal thermistor, thoracoabdominal respiratory effort belts, pulse oximetry, body position, and snoring sensors. Sleep stages and respiratory events were manually scored by an experienced sleep physician.

Respiratory events were scored according to the American Academy of Sleep Medicine (AASM) Scoring Manual update for 2017 (Version 2.4) [15]. Apnea was defined as a ≥90% reduction in airflow lasting at least 10 s. Hypopnea was scored using the AASM recommended rule (Rule 1A), defined as a ≥30% reduction in airflow for at least 10 s accompanied by a ≥3% oxygen desaturation and/or an arousal. The alternative 4% desaturation-only criterion (Rule 1B) was not applied. All polysomnographic recordings were manually scored by an experienced sleep specialist in accordance with AASM standards. Inter-scorer reliability was not assessed because scoring was performed by a single scorer.

The AHI was calculated as the total number of apneas and hypopneas per hour of sleep and was used to classify OSA severity as mild (AHI 5–14.9 events/hour), moderate (AHI 15–29.9 events/hour), or severe (AHI ≥ 30 events/hour). Additional PSG-derived parameters included the oxygen desaturation index (ODI), minimum nocturnal oxygen saturation, total sleep time spent with oxygen saturation below 90%, and snoring index, which were used to assess hypoxic burden and sleep-disordered breathing severity.

#### 2.2.2. Sleepiness Assessment

Subjective daytime sleepiness was evaluated using the Epworth Sleepiness Scale (ESS), a validated self-administered questionnaire consisting of eight items assessing the likelihood of dozing in common daily situations. Each item is scored on a 4-point Likert scale (0–3), yielding a total score ranging from 0 to 24, with higher scores indicating greater daytime sleepiness. An ESS score >10 was considered indicative of excessive daytime sleepiness. The ESS was administered on the night of PSG or during the clinical evaluation preceding PSG [16,17].

#### 2.2.3. Sample Collection and Biochemical Parameters Assessment

Venous blood samples were collected in the morning immediately after overnight polysomnography, following an overnight fasting period. To isolate the serum, the collected blood was centrifuged at 3000 rpm for 10 min. Serum samples were processed within one hour of collection and stored in aliquots at −80 °C to avoid repeated freeze–thaw cycles. All samples were analyzed in a single batch to minimize inter-assay variability. Laboratory personnel performing the ELISA measurements were blinded to participants’ OSA status. Sample order was randomized prior to analysis. Standard biochemical markers were evaluated using an automated clinical chemistry analyzer (Siemens Dimension, Germany) in accordance with routine laboratory techniques.

#### 2.2.4. Assessment of Serum Perilipin-2 (PLIN 2) Concentration

Serum PLIN-2 concentrations were determined using a commercially available sandwich ELISA kit (Human ADRP/Perilipin-2, Cat No: E-EL-H0278, Elabscience, Houston, TX, USA) according to the manufacturer’s instructions. All serum samples were analyzed in duplicate within a single assay batch to minimize inter-assay variability. Laboratory personnel performing the ELISA assays were blinded to the clinical and polysomnographic data of the participants. Prior to analysis, samples were randomized to prevent systematic bias. All samples were analyzed in duplicate within the same assay batch to minimize inter-assay variability. PLIN-2 concentrations were calculated from a standard calibration curve and expressed in ng/mL. The assay demonstrated intra-assay and inter-assay coefficients of variation of 7.4% and 9.1%, respectively, indicating acceptable analytical precision.

### 2.3. Statistical Analysis

Statistical analyses were performed using JASP (version 0.19.3) and R software (version 4.4.2). Continuous variables were tested for normality using the Shapiro–Wilk test. As most variables showed non-normal distribution, data are presented as median (25th–75th percentile). Comparisons among more than two groups were performed using the Kruskal–Wallis test. When a significant overall difference was detected, post hoc pairwise comparisons were conducted using Dunn’s test with Bonferroni correction. Categorical variables were compared using the Chi-square test. Correlations between serum PLIN-2 levels and polysomnographic as well as anthropometric parameters were assessed using Spearman’s rank correlation coefficient. To evaluate the independent association between serum PLIN-2 levels and the presence of OSA, binary logistic regression analysis was performed, including PLIN-2, age, body mass index (BMI), and gender as covariates. The proportional odds assumption underlying the ordinal logistic regression model was evaluated using the test of parallel lines. Results are presented as odds ratios (ORs) with 95% confidence intervals (CIs). For severity-based analyses, ordinal logistic regression was conducted using the proportional odds model. The discriminative performance of serum PLIN-2 for OSA presence and severity was assessed using receiver operating characteristic (ROC) curve analysis. Areas under the curve (AUCs) with 95% confidence intervals were calculated using the DeLong method, and statistical significance was tested against the null hypothesis of AUC = 0.5. Optimal cut-off values were determined using the Youden index. All statistical tests were two-sided, and a *p*-value < 0.05 was considered statistically significant.

Missing data were assessed prior to statistical analysis. The proportion of missing values was low (<5%) and did not demonstrate a systematic pattern. Therefore, analyses were performed using complete-case analysis without imputation. Sensitivity analyses confirmed that exclusion of cases with missing values did not materially affect the results.

## 3. Results

A total of 231 participants were included in the study and categorized as control (*n* = 70), mild OSA (*n* = 60), moderate OSA (*n* = 52), and severe OSA (*n* = 49). Baseline demographic and anthropometric characteristics according to OSA severity are presented in Table 1. Body mass index, waist circumference, hip circumference, waist-to-hip ratio, and neck circumference differed significantly across OSA severity groups (all *p* < 0.001). Post hoc analyses demonstrated significantly higher anthropometric measures in all OSA groups compared with controls. Age did not differ significantly among the severity groups.

Polysomnographic indices according to OSA severity are summarized in Table 2. AHI, oxygen desaturation index (ODI), time spent with oxygen saturation below 90%, snoring index, and ESS scores increased progressively with OSA severity (all *p* < 0.001). Minimum nocturnal oxygen saturation decreased significantly as disease severity increased.

Metabolic and laboratory findings across OSA severity groups are shown in Table 3. Serum glucose, insulin levels, HOMA-IR, total cholesterol, CRP, HbA1c, platelet count, and inflammatory cell counts were significantly higher in patients with OSA compared with controls, with the most pronounced alterations observed in the severe OSA group. Serum creatinine and uric acid levels also showed significant differences across severity categories.

**Figure 1:**
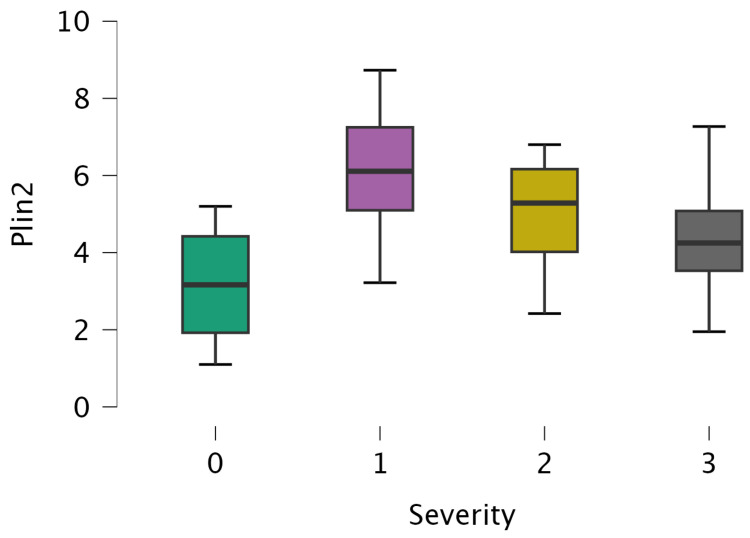
Serum PLIN-2 levels according to OSA severity. Data are presented as median with interquartile range. Group differences were analyzed using the Kruskal–Wallis test followed by Dunn’s post hoc test with Bonferroni correction.

Serum PLIN-2 levels increased progressively with OSA severity (*p* < 0.001; Figure 1). Patients with moderate and severe OSA exhibited significantly higher PLIN-2 levels compared with controls and mild OSA. Spearman correlation analysis revealed that serum PLIN-2 levels were positively correlated with AHI (ρ = 0.597, *p* < 0.001), ODI (ρ = 0.436, *p* < 0.001), ESS score (ρ = 0.604, *p* < 0.001), BMI (ρ = 0.700, *p* < 0.001), waist circumference (ρ = 0.448, *p* < 0.001), and waist-to-hip ratio (ρ = 0.248, *p* < 0.001). PLIN-2 levels were negatively correlated with minimum nocturnal oxygen saturation (ρ = −0.562, *p* < 0.001) (Table 4).

In binary logistic regression analysis adjusted for age, BMI, and gender, serum PLIN-2 levels were independently associated with the presence of OSA (OR > 1, *p* = 0.002), whereas age and gender were not significant predictors. BMI remained a strong independent predictor of OSA presence (*p* < 0.001). The test of parallel lines indicated that the proportional odds assumption was satisfied (*p* > 0.05).

In extended multivariable logistic regression models including HOMA-IR in addition to age, sex, BMI, and PLIN-2, HOMA-IR and BMI remained significant independent predictors of OSA presence (Table 5). However, the association between PLIN-2 and OSA was no longer statistically significant after adjustment for insulin resistance. These findings suggest that the relationship between PLIN-2 and OSA may be partly mediated by metabolic dysfunction.

**Figure 2:**
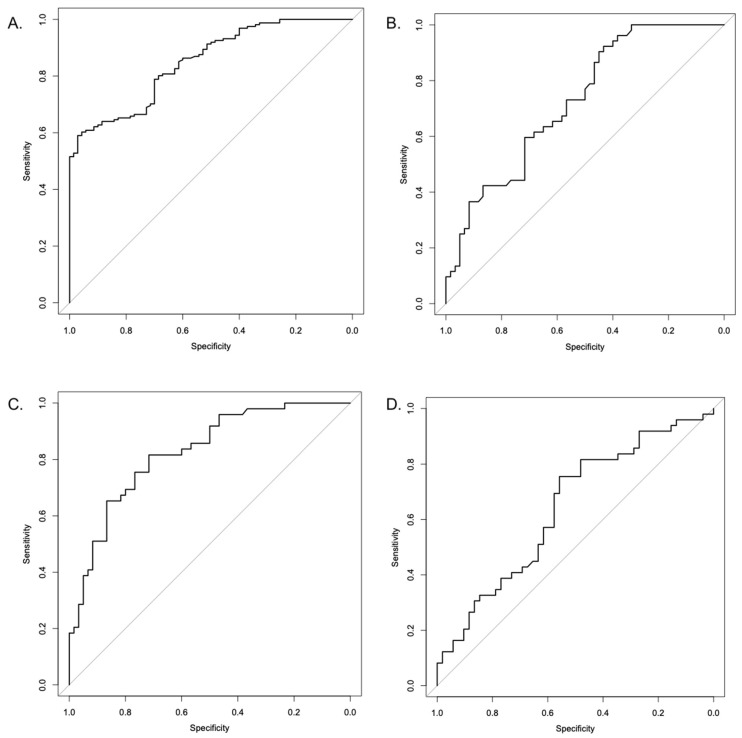
Receiver operating characteristic (ROC) curves of serum PLIN-2 for discrimination of OSA presence and severity. (**A**) Control vs. OSA, (**B**) Mild vs. Moderate OSA, (**C**) Mild vs. Severe OSA, and (**D**) Moderate vs. Severe OSA. Areas under the curve (AUCs) with 95% confidence intervals were calculated using the DeLong method. Optimal cut-off values were determined by the Youden index.

ROC curve analyses demonstrated good discriminative ability of serum PLIN-2 for identifying OSA presence, with an AUC of 0.849 (95% CI 0.800–0.898; *p* < 0.001) (Table 6). Severity-based ROC analyses showed the highest discriminative performance for mild vs. severe OSA (AUC 0.826, 95% CI 0.749–0.902; *p* < 0.001), followed by mild vs. moderate OSA (AUC 0.724, 95% CI 0.632–0.817; *p* < 0.001). Discrimination between moderate and severe OSA was modest (AUC 0.641, 95% CI 0.532–0.749; *p* = 0.011). ROC curves are presented in Figure 2.

**Figure 3:**
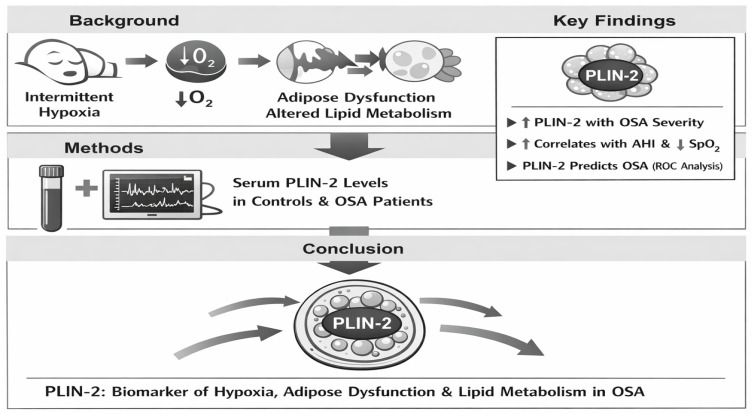
Graphical abstract depicting the role of intermittent hypoxia in adipose tissue dysfunction and elevated serum perilipin-2 (PLIN-2) levels in obstructive sleep apnea.

This mechanistic link between intermittent hypoxia, adipose dysfunction, and the resulting elevation of serum PLIN-2 is summarized in Figure 3.

## 4. Discussion

This study demonstrated that serum PLIN-2 levels are significantly elevated in patients with OSA and increase progressively with disease severity. A key finding is that PLIN-2 remained independently associated with OSA presence after adjustment for major confounders, including age, sex, and BMI. Moreover, PLIN-2 showed strong correlations with hypoxia-related PSG indices (AHI, ODI, and minimum nocturnal oxygen saturation), supporting the concept that PLIN-2 may reflect hypoxic–metabolic burden in OSA. However, given the modest sensitivity at the optimal ROC threshold, PLIN-2 should be interpreted as an adjunct biomarker rather than a stand-alone screening test. Although serum PLIN-2 levels were independently associated with OSA presence and severity, the cross-sectional design of this study precludes any inference of causality. Therefore, elevated PLIN-2 should be interpreted as a biological correlation of OSA-related metabolic stress rather than a causal factor in disease pathogenesis.

Consistent with prior reports, OSA patients exhibited significantly higher BMI, waist circumference, hip circumference, waist-to-hip ratio, and neck circumference compared with controls, emphasizing the close relationship between central adiposity and OSA severity [18,19]. In our cohort, anthropometric measures increased in parallel with disease severity, reinforcing that obesity-related anatomical and metabolic factors strongly contribute to upper airway collapsibility. Data show that visceral adiposity indices align with PSG findings, highlighting that neck circumference serves as a critical factor in increasing upper airway resistance and elevating AHI scores [20,21]. Notably, age did not differ across groups, suggesting that in this cohort, metabolic and anatomical determinants may predominate over aging-related effects. This observation supports the clinical relevance of early anthropometric risk assessment in young and middle-aged individuals [22,23].

Our PSG results confirm a graded relationship between OSA severity and markers of intermittent hypoxia. AHI and ODI increased, whereas minimum nocturnal oxygen saturation decreased progressively with severity, consistent with intermittent hypoxia as a core driver of systemic sequelae [24,25,26]. A cohort study in 2021 showed that ODI independently worsened cardiovascular risk profiles [24]. In addition, the parallel increase in ESS with severity suggests that subjective daytime sleepiness tracks objective respiratory impairment. A recent review [27] emphasized that hypoxemia burden (e.g., T90) is among the strongest predictors of metabolic dysfunction, underscoring that hypoxia metrics beyond AHI may provide important prognostic information [28].

Our findings also reinforce OSA as a systemic disorder associated with impaired glucose homeostasis, dyslipidemia, and inflammation. Severe OSA was characterized by the most pronounced elevations in insulin, HOMA-IR, and HbA1c, supporting intermittent hypoxia as a pathophysiological contributor to insulin resistance. A large study in 2022 reported that chronic hypoxia promotes adipose tissue dysfunction and increases the risk of T2DM independently [29]. We also observed significant increases in CRP, platelet count, and inflammatory cell indices, compatible with persistent low-grade inflammation. Elevated uric acid and differences in creatinine across severity categories may reflect oxidative stress and renal microvascular vulnerability described in OSA populations [30,31,32,33,34]. Together, these changes support a multi-organ metabolic–inflammatory phenotype accompanying severe disease.

PLIN-2 is a lipid droplet surface protein involved in triglyceride storage and protection from lipolysis [35]. Intermittent hypoxia central to OSA can disrupt lipid metabolism and promote lipid droplet remodeling [36]. The most novel observation in our study is the progressive rise in serum PLIN-2 levels with increasing OSA severity, together with strong correlations with AHI, ODI, ESS, and anthropometric indices.

These results align with evidence that PLIN-2 is elevated in metabolic and inflammatory states such as obesity, atherosclerosis, and NAFLD [37,38,39]. Conte et al. [37] reported that circulating PLIN-2 is associated with fat mass and systemic inflammation; while sex-related differences have been noted in general populations, our adjusted models indicate that in the context of OSA, PLIN-2 is associated with disease presence independent of age and sex. This may suggest that intermittent hypoxia and oxidative stress in OSA can outweigh baseline physiological variability.

Importantly, the reviewer’s point regarding clinical utility is well taken although the overall AUC indicates good discrimination (AUC = 0.849), the optimal cut-off yielded modest sensitivity, limiting PLIN-2 as a screening marker. Furthermore, BMI was a stronger predictor than PLIN-2 in regression models. Therefore, the clinically relevant question is what PLIN-2 adds beyond BMI. In our study, PLIN-2 remained associated with OSA after BMI adjustment, implying that PLIN-2 may capture hypoxia-related lipid droplet dysregulation and metabolic stress not fully represented by anthropometric indices alone. Nonetheless, independent association does not automatically imply incremental prediction, and future work should formally assess additive value using model comparison (e.g., ΔAUC, likelihood ratio tests, calibration, and reclassification metrics) and evaluate whether PLIN-2 improves risk stratification within BMI strata or specific metabolic phenotypes [40].

Accordingly, PLIN-2 should be framed as a mechanistic/phenotyping biomarker that may complement PSG and clinical assessment rather than replace them. This may be particularly relevant for identifying OSA patients with higher cardiometabolic risk profiles, although prospective validation is required [41,42].

### 4.1. Mediation by Insulin Resistance

An important observation in the present study is that the association between serum PLIN-2 and OSA lost statistical significance after adjustment for HOMA-IR in extended multivariable models, whereas HOMA-IR remained a strong independent predictor. This pattern suggests that the relationship between PLIN-2 and OSA may be at least partly mediated by insulin resistance. Rather than diminishing the relevance of PLIN-2, this finding provides mechanistic insight. Intermittent hypoxia is known to induce adipose tissue dysfunction, oxidative stress, and impaired glucose metabolism, which collectively promote insulin resistance. Given its role in lipid droplet stabilization and intracellular triglyceride storage, PLIN-2 may represent a downstream marker of hypoxia-driven metabolic remodeling rather than a primary pathogenic factor in OSA. Although a formal mediation analysis was not performed, the attenuation of the PLIN-2 effect after inclusion of HOMA-IR supports the hypothesis that insulin resistance may lie on the causal pathway linking intermittent hypoxia to altered lipid droplet biology. Future longitudinal studies incorporating structured mediation modeling are warranted to clarify temporal sequencing and causal relationships.

### 4.2. Unexpected Findings and Possible Explanations

Several results showed counterintuitive patterns, including lower BMI and/or ESS values in severe OSA and a decreasing uric acid trend with increasing severity. These findings likely reflect clinical heterogeneity and residual confounding rather than protective effects. OSA severity is not determined by BMI alone; severe disease may occur at lower BMI due to upper-airway anatomical susceptibility, ventilatory control instability, or preferential central fat distribution, for which waist and neck circumference may be more informative. ESS is subjective and may not track physiological severity, as symptom perception varies and severe OSA can present predominantly with cardiometabolic manifestations. Uric acid is influenced by renal handling, hydration, diet, and medication use (e.g., diuretics or urate-lowering therapy), which may obscure its relationship with hypoxic burden.

Unlike conventional inflammatory markers such as CRP or uric acid—which primarily reflect systemic inflammation and oxidative stress PLIN-2 is directly involved in lipid droplet biology and intracellular triglyceride storage. While adipokines and inflammatory cytokines provide insight into generalized metabolic and inflammatory activation in OSA, PLIN-2 may offer more specific information regarding hypoxia-driven lipid droplet remodeling and adipocyte metabolic stress [8,35]. Therefore, PLIN-2 should not be interpreted merely as a nonspecific marker of obesity or inflammation, but rather as a potential indicator of altered lipid storage dynamics associated with intermittent hypoxia in OSA.

### 4.3. Limitations and Strengths

Despite its novel findings, this study has several limitations. First, the cross-sectional, single-center design precludes causal inference and may limit generalizability. Second, the control group was intentionally selected to be free of major comorbidities and medications affecting lipid metabolism, which ensured a clearly defined reference but may have produced a relatively metabolically healthier comparator group; thus, some between-group differences may partly reflect broader metabolic disparities rather than OSA-specific effects. Third, although standardized PSG scoring and laboratory procedures were applied, residual measurement variability and pre-analytical factors (e.g., sample handling and storage) cannot be fully excluded, and unmeasured confounders (e.g., detailed medication use, dietary factors, subclinical inflammation) may persist. Finally, mechanistic pathways linking intermittent hypoxia to PLIN-2 regulation were not directly examined. Future multicenter, longitudinal, and interventional studies—including metabolically matched controls and treatment-response analyses—are warranted to clarify temporal relationships and the independent contribution of OSA to circulating PLIN-2 levels.

In contrast, a key strength of the study is that it is the first clinical investigation to evaluate serum PLIN-2 in OSA using gold-standard attended polysomnography in a well-phenotyped cohort. The integration of anthropometric, metabolic, inflammatory, and PSG measures supported by robust regression and ROC analyses strengthens the clinical and biological relevance of the findings.

### 4.4. Clinical Implications

Serum PLIN-2 was associated with OSA severity and hypoxia-related polysomnographic indices, suggesting that it may reflect hypoxic–metabolic stress and adipose tissue dysfunction in OSA. However, given the cross-sectional design and the modest sensitivity at the optimal ROC threshold, PLIN-2 should be considered an adjunct biomarker for phenotyping and cardiometabolic risk stratification rather than a stand-alone screening tool. Future multicenter longitudinal and interventional studies are needed to determine whether PLIN-2 levels change with effective OSA treatment (e.g., CPAP) and whether PLIN-2 provides incremental prognostic value beyond established predictors. In addition, mechanistic investigations and detailed upper-airway phenotyping (e.g., imaging-based fat distribution or drug-induced sleep endoscopy) are warranted to clarify biological pathways and potential differences across anatomical OSA subtypes.

## 5. Conclusions

This study supports the concept that OSA is accompanied by progressive deterioration in anthropometric, polysomnographic, metabolic, and inflammatory profiles. Serum PLIN-2 increases with OSA severity and is associated with hypoxia-related indices, suggesting a link between intermittent hypoxia and lipid droplet-related metabolic dysregulation. While PLIN-2 shows promise as a biological correlate of hypoxic–metabolic burden, further longitudinal and multicenter studies are needed to determine its treatment responsiveness and incremental clinical value beyond established predictors such as BMI.

## Figures and Tables

**Table 1:** Baseline characteristics according to OSA severity.

Variable	Control (*n* = 70)	Mild (*n* = 60)	Moderate (*n* = 52)	Severe (*n* = 49)
Age (years)	44 (41–50.75)	46.5 (44–49)	46 (43–50)	47 (40–55)
Gender (Female (*n*)/Male (*n*))	39/31	30/30	20/32	23/26
BMI (kg/m^2^)	23.69 (22.02–24.98)	27.43 (24.44–29.67) a ***	33.79 (31.13–36.59) a ***	32.80 (31.13–35.52) a ***, b ***, c ***
Waist circumference (cm)	85 (75–90.75)	112 (105.5–117.25) a ***	115 (108–125) a ***	109 (100–118) a ***
Hip circumference (cm)	96.5 (92–103.75)	120 (113–125.25) a ***	124 (115.5–131.25) a ***	122 (115–133) a ***
Waist/Hip ratio	0.87 (0.83–0.89)	0.93 (0.89–0.96) a ***	0.93 (0.88–1.03) a ***	0.90 (0.82–0.94)
Neck circumference (cm)	36 (33.25–37)	40 (36.5–44) a ***	40 (38–41) a ***	39 (35–41) a ***

Data are presented as median (25th–75th percentile). Overall comparisons were performed using the Kruskal–Wallis test. Post hoc pairwise comparisons were conducted using Dunn’s test with Bonferroni correction. a *** *p* < 0.001 vs. control; b *** *p* < 0.001 vs. mild OSA; c *** *p* < 0.001 vs. moderate OSA.

**Table 2:** Polysomnographic parameters according to OSA severity.

Variable	Control (*n* = 70)	Mild (*n* = 60)	Moderate (*n* = 52)	Severe (*n* = 49)
AHI (events/h)	1 (1–2)	19 (11–23) a ***	19.8 (17.775–22.475) a ***, b ***	50.5 (37.75–78) a ***, b ***
ODI (events/h)	0 (0–0)	21.5 (15–30.25) a ***	16 (10.45–25.125) a ***	31 (14–55) a ***
Minimum SaO_2_ (%)	97 (96–98)	79 (75.75–83.25) a ***	84.470 (80.987–87) a ***, b *	86 (80–90) a ***, b ***
Overnight SaO_2_ < 90%	1 (1–1)	49.5 (32–70.25) a ***	43.5 (24–100) a ***	124 (71–196) a ***, b **, c *
Snoring index	1 (1–1)	33 (25–42) a ***	94 (27.25–133) a ***	229 (79–302) a ***, b ***
Epworth score	0 (0–0)	3 (1–7) a ***	10 (5.75–11) a ***, b ***	17 (15–19) a ***, b ***, c **

Data are presented as median (25th–75th percentile). Overall comparisons were performed using the Kruskal–Wallis test. Post hoc pairwise comparisons were conducted using Dunn’s test with Bonferroni correction. a means vs. control; b means vs. mild OSA; c means vs. moderate OSA; *: *p* < 0.05, **: *p* < 0.01 and ***: *p* < 0.001.

**Table 3:** Metabolic and laboratory parameters according to OSA severity.

Variable	Control (*n* = 70)	Mild (*n* = 60)	Moderate (*n* = 52)	Severe (*n* = 49)
Glucose (mg/dL)	91 (84.25–95)	106 (93–178.25) a ***	118 (98.5–181.25) a ***	170 (133–198) a ***, b **, c *
Insulin (µIU/mL)	8.4 (7.43–9.3)	17.3 (15.0–19.6) a ***	19.25 (15.18–22.95) a ***	21.1 (16.2–25.3) a ***
HOMA-IR	1.85 (1.6–2.1)	4.7 (3.7–7.53) a ***	5.4 (4.3–8.15) a ***	7.9 (5.9–10.8) a ***, b **
Creatinine (mg/dL)	0.73 (0.63–0.86)	0.76 (0.65–0.94)	0.88 (0.72–1.06) a **	0.89 (0.63–1.05) a *
Uric acid (mg/dL)	3.65 (3.10–4.08)	3.20 (2.60–4.30) a *	3.61 (3.10–4.74)	4.05 (3.38–5.55) b ***
LDL (mg/dL)	100 (88–111.75)	121.5 (97.75–151) a ***	112 (81–151.75)	117 (85–139)
HDL (mg/dL)	50.5 (43–60)	47.5 (40–58.25)	44.5 (38.75–48) a **	46 (40–60)
Triglycerides (mg/dL)	112.5 (75–142.25)	104 (91.75–121.25)	118.5 (93–144)	110 (89–121)
Total cholesterol (mg/dL)	179 (163.5–189)	200.5 (176–230.5) a ***	214 (174.3–246.3) a ***	236 (186–272) a ***
CRP (mg/L)	1.46 (0.81–1.94)	5.12 (2.44–13.84) a ***	8.38 (3.39–9.82) a ***	7.18 (4.19–9.91) a ***
HbA1c (%)	5.4 (5.3–5.78)	6.6 (6.0–7.45) a ***	6.6 (5.9–7.43) a ***	6.8 (6.0–7.6) a ***
Platelet (×10^3^/µL)	272 (225.5–293.8)	303 (250.8–350.8)	297 (262.5–340) a *	314 (253–366) a **
Lymphocyte (×10^3^/µL)	2.10 (1.90–2.40)	2.85 (2.28–3.40) a *	5.90 (5.70–6.43) a ***, b ***	5.30 (4.10–6.00) a ***, b ***, c *
Neutrophil (×10^3^/µL)	3.95 (3.37–4.55)	5.23 (4.01–7.08) a ***	5.46 (3.30–7.73) a ***	7.38 (5.90–10.25) a ***, b **, c ***
Monocyte (×10^3^/µL)	0.42 (0.35–0.49)	0.60 (0.45–0.86) a ***	0.72 (0.42–1.16) a ***	0.82 (0.52–1.17) a ***

Data are presented as median (25th–75th percentile). Overall comparisons were performed using the Kruskal–Wallis test. Post hoc pairwise comparisons were conducted using Dunn’s test with Bonferroni correction. a means vs. control; b means vs. mild OSA; c means vs. moderate OSA; *: *p* < 0.05, **: *p* < 0.01 and ***: *p* < 0.001.

**Table 4:** Spearman correlation analysis between serum PLIN-2 levels and clinical, anthropometric, and polysomnographic parameters.

Variable	Spearman r	*p*-Value
AHI	0.597	1.03 × 10^−23^
ODI	0.436	3.83 × 10^−12^
Minimum SaO_2_	–0.562	1.33 × 10^−20^
SaO_2_ < 90%	0.387	1.13 × 10^−9^
Epworth score	0.604	2.25 × 10^−24^
BMI	0.700	2.83 × 10^−35^
Waist circumference	0.448	8.09 × 10^−13^

**Table 5:** Extended multivariate logistic regression analysis for the presence of OSA including metabolic parameters.

Variable	β (SE)	Adjusted OR	95% CI	*p*-Value
PLIN-2	−0.118 (0.590)	0.89	0.28–2.83	0.842
Age (years)	0.064 (0.113)	1.07	0.85–1.33	0.573
BMI (kg/m^2^)	0.962 (0.275)	2.62	1.53–4.49	<0.001
Gender	−1.498 (1.410)	0.22	0.01–3.54	0.288
HOMA-IR	3.216 (0.950)	24.94	3.88–160.60	<0.001

Model statistics: Deviance = 20.193; AIC = 32.193; BIC = 52.848; Δχ^2^ = 263.202, *p* < 0.001; McFadden R^2^ = 0.929; Nagelkerke R^2^ = 0.962; Tjur R^2^ = 0.951; Cox & Snell R^2^ = 0.680.

**Table 6:** ROC analysis of serum PLIN-2 for OSA presence and severity discrimination.

Comparison	AUC (95% CI)	*p*-Value (Two-Sided)	Optimal Cut-Off (Youden)	Sensitivity (%)	Specificity (%)
Control vs. OSA (Disease 0 vs. 1)	0.849 (0.800–0.898)	1.13 × 10^−44^	4.92	59.0	97.1
Mild vs. Moderate OSA (1 vs. 2)	0.724 (0.632–0.817)	1.94 × 10^−6^	6.55	92.3	43.3
Mild vs. Severe OSA (1 vs. 3)	0.826 (0.749–0.902)	7.27 × 10^−17^	5.41	81.6	71.7
Moderate vs. Severe OSA (2 vs. 3)	0.641 (0.532–0.749)	0.0112	5.12	75.5	55.8

ROC analyses were performed using the pROC package. The significance of AUC values was assessed against the null hypothesis AUC = 0.5 using DeLong variance estimates (two-sided). Optimal cut-off values were determined by the Youden index.

## Data Availability

The data that support the findings of this study are available on request from the corresponding author. The data are not publicly available due to privacy or ethical restrictions.
